# Improving hand hygiene compliance in child daycare centres: a randomized controlled trial

**DOI:** 10.1017/S0950268816000911

**Published:** 2016-05-19

**Authors:** T. P. ZOMER, V. ERASMUS, C. W. LOOMAN, E. F. VAN BEECK, A. TJON-A-TSIEN, J. H. RICHARDUS, H. A. C. M. VOETEN

**Affiliations:** 1 Department of Infectious Disease Control, Municipal Public Health Service Rotterdam-Rijnmond, Rotterdam, The Netherlands; 2 Department of Public Health, Erasmus MC, University Medical Centre Rotterdam, Rotterdam, The Netherlands

**Keywords:** Child daycare, guidelines, hand hygiene, infectious disease control, intervention

## Abstract

Gastrointestinal and respiratory infections in children attending daycare centres (DCCs) are common and compliance with hand hygiene (HH) guidelines to prevent infections is generally low. An intervention was developed to increase HH compliance and reduce infections in DCCs. The objective of this paper was to evaluate the effectiveness of this intervention on HH compliance. The intervention was evaluated in a two-arm cluster randomized controlled trial in 71 DCCs in The Netherlands. Thirty-six DCCs received the intervention including: (1) HH products; (2) training about HH guidelines; (3) two team training sessions aimed at goal setting and formulating HH improvement activities; and (4) reminders and cues for action (posters/stickers). Intervention DCCs were compared to 35 control DCCs that continued usual practice. HH compliance of caregivers and children was observed at baseline and at 1, 3 and 6 months follow-up. Using multilevel logistic regression, odds ratios (ORs) with 95% confidence intervals (CIs) were obtained for the intervention effect. Of 795 caregivers, 5042 HH opportunities for caregivers and 5606 opportunities for supervising children's HH were observed. At 1 month follow-up caregivers' compliance in intervention DCCs was 66% *vs.* 43% in control DCCs (OR 6·33, 95% CI 3·71–10·80), and at 6 months 59% *vs.* 44% (OR 4·13, 95% CI 2·33–7·32). No effect of the intervention was found on supervising children's HH (36% *vs.* 32%; OR 0·64, 95% CI 0·18–2·33). In conclusion, HH compliance of caregivers increased due to the intervention, therefore dissemination of the intervention can be considered.

## INTRODUCTION

Attending child daycare centres (DCCs) has been associated with increased risk of acquiring gastrointestinal and respiratory infections [1–3]. These infections can cause parental stress, secondary transmission, healthcare costs, and costs for parental work absence [4–7]. Hand hygiene (HH) is known to be an effective measure to prevent infections [8, 9]. However, compliance with HH guidelines in DCCs is generally low [10]. Although several HH interventions have been developed to reduce infections in children attending DCCs [11–18], these interventions show varying results [19] and are not developed according to a stepwise behavioural approach taking into account the determinants that underlie HH behaviour [20].

Our previous research showed that environmental determinants, such as the availability of paper towels, are associated with caregivers' HH compliance in DCCs [10]. In addition, we found that the following sociocognitive determinants are associated with HH compliance of DCC caregivers: knowledge and awareness of HH guidelines, perceived importance of performing HH, perceived behavioural control (i.e. perceived ease or difficulty of performing the behaviour) and habit [21]. We developed an intervention targeting these determinants aiming to increase compliance with HH guidelines and reduce gastrointestinal and respiratory infections in children attending DCCs. We assessed both HH compliance and incidence of infections as outcome measures. HH compliance as outcome measure provides insight into a more direct effect of the intervention and might explain the variation in effectiveness of previous HH intervention studies assessing disease incidence. In this paper we assess the effectiveness of our intervention on improving HH compliance. The effectiveness on disease incidence is reported separately [22].

## METHODS

A cluster randomized controlled trial of a HH intervention was performed in DCCs in the regions of Rotterdam-Rijnmond, Gouda and Leiden in The Netherlands between September 2011 and April 2012. DCCs were randomized, stratified for DCC size and urbanicity [23]. In our previous study on determinants of caregivers' HH compliance, 122 DCCs participated [10, 21]. These DCCs were contacted for participation in the trial. Sample size calculation showed that 35 intervention DCCs and 35 control DCCs were needed [23].

The intervention consisted of four components [23]. First, the following HH products were provided free of charge with refills for 6 months: dispensers for paper towels, soap, alcohol-based hand sanitizer and hand cream. Second, training was given to educate DCC caregivers about the Dutch national HH guidelines. This included a hand-washing exercise using UV Glow Cream (Deb Benelux Inc.) and an information booklet outlining the content of the training. Third, two team training sessions were given aimed at goal setting and formulating specific HH improvement activities. These were based on similar HH training sessions developed for Dutch hospitals [24]. Fourth, reminders and cues for action were provided for both caregivers and children (i.e. posters and stickers). Due to budget restrictions, the HH products were provided for two groups of the DCC, even if the DCC had more than two groups in total. The other intervention components were provided for all DCC groups and staff members.

Intervention DCCs were compared to control DCCs that continued usual practice. The primary outcome measure was observed HH compliance of caregivers. Compliance was defined as the number of HH actions divided by the total number of opportunities for which HH was indicated. According to Dutch national guidelines, HH is mandatory for caregivers before touching/preparing food, before caregivers themselves ate or assisted children with eating, and before wound care; and after diapering, after toilet use/wiping buttocks, after caregivers themselves coughed/sneezed/wiped their own nose, after contact with body fluids (e.g. saliva, vomit, urine, blood, or mucus when wiping children's noses), after wound care, and after hands were visibly soiled [25]. For these HH indications it was observed whether or not HH was performed. As observations could not take place in the caregivers' lavatory, HH of caregivers after toilet use was not observed. HH was defined as washing hands with water and soap followed by hand drying, or using an alcohol-based hand sanitizer (which could only be used if hands were not visibly soiled).

Although the primary outcome measure was HH compliance of caregivers, it was also observed whether caregivers supervised children in washing their hands before eating/preparing food, after toilet use, after playing outside, and after hands were visibly soiled, as indicated in the HH guidelines [25]. Children had to wash their hands with water and soap followed by hand drying. For babies and toddlers who could not wash their hands themselves yet, caregivers could perform HH by using a wet cloth [25].

Compliance was assessed with direct unobtrusive observation by trained observers before the start of the intervention (T0) and 1 (T1), 3 (T3), and 6 (T6) months after start of the intervention. In total, 13 observers were trained aiming for an inter-rater reliability above 75%. Data collection followed phased implementation of the intervention [23]. After observing baseline compliance (T0), intervention DCCs received the HH products, posters/stickers and training regarding the HH guidelines; after this HH compliance was observed again (T1), and once more after each of both team training sessions (T3 and T6). At each measurement, the aim was to observe three caregivers for 2 h in two participating groups per DCC. One observer observed one caregiver at a time, as well as the children under his/her care. Data were collected using the World Health Organization HH observation method [26], adapted for use in child DCCs. At 6 months follow-up, it was also observed whether the dispensers provided as part of the intervention were (still) in use. After the last observations, a survey was conducted among caregivers in intervention DCCs concerning their exposure to the different intervention components.

Data were analysed using SPSS v. 19 (SPSS Inc., USA) and R v. 2.12.2 (https://cran.r-project.org). Analyses were performed including all intervention DCCs irrespective of whether they used the HH products, posters/stickers or obtained all training sessions (intention-to-treat analyses). First, baseline characteristics were compared. Second, compliance at baseline and total follow-up (T1, T3 and T6 together) was calculated, as well as compliance for the separate follow-up measurements (T1, T3 and T6 separate). For 6 months follow-up (T6), compliance was calculated for each of the HH indications. Multilevel regression analyses were performed to correct for clustering of the data within DCCs and within caregivers. Using multilevel logistic regression analyses for total follow-up and for each separate follow-up measurement, odds ratios (ORs) with 95% confidence intervals (CIs) were obtained for the intervention effect (i.e. intervention status of the DCC: intervention *vs.* control), corrected for confounders that showed significant differences at baseline between intervention and control DCCs. Because the type of activity for which HH was indicated had previously been shown to be an important determinant of caregivers' HH [10], this was also included as a confounder. Additional analyses were performed to correct for baseline compliance. For this we calculated the intervention effect as the interaction between intervention status of the DCC (i.e. intervention *vs.* control) and follow-up measurement (i.e. baseline *vs.* T1, baseline *vs.* T3, baseline *vs.* T6, baseline *vs.* total follow-up).

Ethical approval was waived by the Medical Ethics Committee of the Erasmus University Medical Center in Rotterdam (MEC-2011-256).

## RESULTS

Of 122 DCCs, 71 DCCs participated in the trial (response rate 58%). After randomization, there were 36 intervention and 35 control DCCs. At baseline and 1 month after start of the intervention, all 71 DCCs participated. Three months after start of the intervention, one control DDC was lost to follow-up, and 6 months after start of the intervention two more control DCCs were lost to follow-up. In total, 795 caregivers and 5042 HH opportunities were observed. In addition, 5606 opportunities were observed for supervising children's HH. The inter-rater reliability of the observers was ⩾74%.

Comparison of baseline characteristics of intervention and control DCCs demonstrated that in intervention DCCs, age group (0–1, 2–3, 0–4 years) significantly differed from control DCCs (Table 1). This variable was therefore included in further analyses as a confounder. None of the other baseline characteristics were significantly different between intervention and control DCCs (Table 1).

**Table 1.** Comparison of baseline characteristics (N = 71 DCCs)

	Intervention DCCs	Control DCCs	
DCC characteristics	(*N* = 36)	(*N* = 35)	*P* value
Size (large, having ⩾46 children per day)	53%	51%	0·91
Degree of urbanicity			0·84
Highly urban	58%	63%	
Urban	22%	23%	
Slightly/non-urban	19%	14%	
Region			
Rotterdam-Rijnmond	67%	66%	0·47
Gouda	14%	6%	
Leiden	19%	29%	
Certification (certified)	44%	41%	0·83
Age group, years*			0·03
0–1	21%	31%	
2–3	13%	24%	
0–4	67%	44%	
Number of towel facilities for caregivers per group*	1·63	1·54	0·68§
Type of towel facilities for caregivers in the group*			0·14
Only paper towels	25%	35%	
Only fabric towels	44%	48%	
Both fabric and paper towels	31%	17%	
Number of soap facilities for caregivers per group*	1·55	1·52	0·90§
Type of soap facilities for caregivers in the group*			0·66
Only soap dispensers	14%	11%	
Only soap pumps	70%	77%	
Soap dispensers combined with soap pumps	16%	12%	
Alcohol-based hand sanitizer for caregivers in the group (available)*	67%	59%	0·30
Number of towel facilities for children per group†	0·98	1·00	0·93§
Type of towel facilities for children in the group†			1·00
Only paper towels	46%	46%	
Only fabric towels	44%	44%	
No towel facilities in reach of children	11%	10%	
Number of soap facilities for children per group†	0·84	0·75	0·62§
Type of soap facilities for children in the group†			0·20
Only soap dispensers	42%	25%	
Only soap pumps	35%	48%	
No soap facilities in reach of children	23%	27%	
Number of children per caregiver‡	5·2	5·1	0·63||

DCC, Daycare centre.

* *N* = 72 intervention groups and 70 control groups.

† *N* = 57 intervention groups and 48 control groups (groups with children aged 0–2 years were excluded).

‡ *N* = 105 intervention caregivers and 102 control caregivers.

§ Estimated with Poisson regression.

|| Estimated with independent *t* test.

All 36 intervention DDCs received training on HH guidelines. Of 36 intervention DCCs, two DCCs did not use any of the provided HH products during the study period. Another two DCCs did not receive any of the team training sessions. At 6 months follow-up, 94% (33/35) of intervention DCCs used the paper towel dispensers in at least one of the two groups, 89% (31/35) used the soap dispensers, 86% (30/35) used the dispensers with alcohol-based hand sanitizer and 45% (13/29) used the dispensers with hand cream. At 6 months follow-up, in 19% of intervention DCCs (7/36), neither posters nor stickers of the intervention were used, in 83% (29/35) the posters were used in at least one of two groups, and in 74% (26/35) the stickers were used. The response rate to the questionnaire on exposure to the intervention was 50% (274/546). Of 274 caregivers, 21% (54/261) attended none of the training sessions, 25% (66/261) attended one training session, 29% (75/261) attended two training sessions and 25% (66/261) attended all three sessions. Of 274 caregivers, 77% (202/262) received the information booklet of the training session on HH guidelines.

### HH compliance of caregivers

**Fig. 1.**
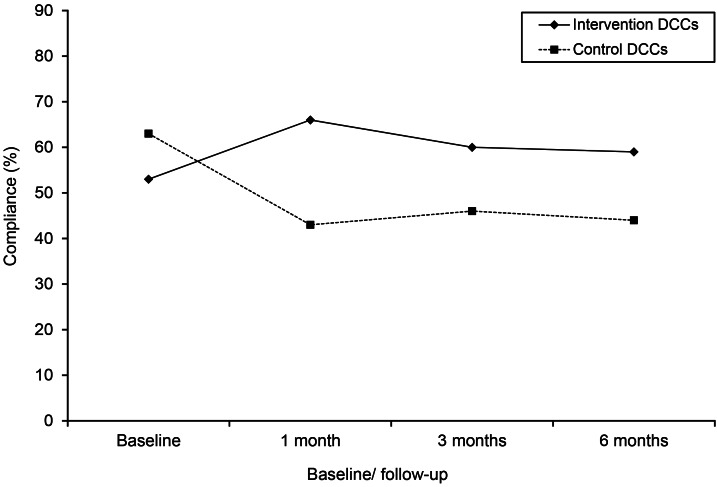
Effect of the intervention on caregivers' compliance with hand hygiene guidelines in child daycare centres (DCCs) measured at baseline and 1, 3 and 6 months after intervention start.

Figure 1 shows that caregivers' HH compliance at baseline was lower in intervention DCCs than in control DCCs. During follow-up, compliance in intervention DCCs was higher than in control DCCs, although the effect of the intervention seemed to wane slightly.

Compliance at baseline was not significantly different between intervention and control DCCs (respectively 53% *vs.* 63%; OR 0·62, 95% CI 0·38–1·02) (Table 2). Overall compliance during total follow-up (i.e. taking T1, T3 and T6 together) in intervention DCCs was 62% (1243/2005) *vs.* 44% (812/1850) in control DCCs. Correcting for type of activity for which HH was indicated, age group, and clustering of the data within caregivers and within DCCs, the OR was 2·69 (95% CI 1·88–3·86). The OR was 4·65 (95% CI 2·99–7·25) when also taking into account baseline compliance. One month after the start of the intervention, compliance in intervention DCCs was 66% (459/692) *vs.* 43% (273/640) in control DCCs. This difference was significant, correcting for type of activity for which HH was indicated, age group, and clustering of the data within caregivers and within DCCs (OR 3·53, 95% CI 2·23–5·61). Three months after the start of the intervention, compliance was 60% (392/649) in intervention DCCs *vs.* 46% (273/600) in control DCCs (OR 2·45, 95% CI 1·58–3·80). Six months after the intervention start, compliance in intervention DCCs was 59% (392/664) *vs.* 44% (266/610) in control DCCs (OR 2·49, 95% CI 1·39–4·46). When also taking into account baseline compliance the OR for the intervention effect after 6 months was 4·13 (95% CI 2·33–7·32).

**Table 2.** Effect of the intervention on compliance with hand hygiene (HH) guidelines in child daycare centres (DCCs) measured at baseline and 1, 3 and 6 months after start of the intervention

Baseline/ follow-up	Intervention status of the DCC	No. of DCCs	No. of caregivers observed	No. of HH opportunities for caregivers	Compliance of caregivers (%)	OR* (95% CI)	Baseline corrected OR† (95% CI)	No. of HH opportunities for children	Compliance of children (%)	OR* (95% CI)	Baseline corrected OR† (95% CI)
Baseline	Intervention	36	105	623	53	0·62 (0·38–1·02)	_	583	51	4·24 (1·38–12·96)	_
	Control	35	102	564	63	Ref.		478	38	Ref.	
1 month follow-up	Intervention	36	105	692	66	3·53 (2·23–5·61)	6·33 (3·71–10·80)	738	39	1·31 (0·38–4·60)	0·30 (0·08–1·16)
	Control	35	97	640	43	Ref.	Ref.	637	42	Ref.	Ref.
3 months follow-up	Intervention	36	101	649	60	2·45 (1·58–3·80)	4·08 (2·37–7·02)	770	44	1·40 (0·54–3·63)	0·83 (0·20–3·38)
	Control	34	97	600	46	Ref.	Ref.	841	39	Ref.	Ref.
6 months follow-up	Intervention	36	99	664	59	2·49 (1·39–4·46)	4·13 (2·33–7·32)	854	36	3·03 (1·02–9·03)	0·64 (0·18–2·33)
	Control	32	89	610	44	Ref.	Ref.	705	32	Ref.	Ref.
Total follow-up	Intervention	36	305	2005	62	2·69 (1·88–3·86)	4·65 (2·99–7·25)	2362	40	1·62 (0·77–3·42)	0·78 (0·25–2·44)
	Control	35	283	1850	44	Ref.	Ref.	2183	37	Ref.	Ref.

OR, Odds ratio; CI, confidence interval.

* Difference between intervention and control DCCs corrected for the type of activities for which HH was indicated, age group (i.e. 0–1, 2–3 and 0–4 years), and clustering of the data within caregivers and within DCCs.

† Interaction effect of intervention status of the DCC and baseline/follow-up measurement corrected for the type of activities for which HH was indicated, age group (i.e. 0–1, 2–3 and 0–4 years), and clustering of the data within caregivers and within DCCs.

### HH compliance of children

Children's HH compliance at baseline in intervention DCCs was significantly higher than in control DCCs (respectively, 51% *vs.* 38%; OR 4·24, 95% CI 1·38–12·96) (Table 2). Children's compliance during follow-up (i.e. taking T1, T3 and T6 together) in intervention DCCs was 40% (936/2362) *vs.* 37% (811/2183) in control DCCs. Corrected for type of activity for which HH was indicated, age group, and clustering of the data within caregivers and within DCCs, there was no significant difference (OR 1·62, 95% CI 0·77–3·42). When also taking into account baseline compliance the difference remained non-significant (OR 0·78, 95% CI 0·25–2·44).

Comparison of the different types of activities for which HH was indicated showed that at 6 months follow-up there was a significant increase in HH compliance (taking into account baseline) after toilet and diapering activities (OR 4·49, 95% CI 2·23–9·05) and after contact with body fluids (OR 4·88, 95% CI 1·77–13·44) (Table 3). Of toilet and diapering activities, the largest difference in HH compliance between intervention and control DCCs was 46% after changing a wet diaper when a child was standing. For activities with body fluid contact, the largest difference was 47% after caregivers coughed/sneezed/wiped their own nose (Table 3). The increase in caregivers' HH compliance before eating and food-handling activities was not significant (OR 1·95, 95% CI 0·76–5·00) (Table 3).

**Table 3.** Effect of the intervention on the compliance with each specific hand hygiene (HH) indication outlined in the Dutch national guidelines for child daycare centres (DCCs), measured at baseline and 6 months after start of the intervention

	Follow-up compliance at 6 months							
	Intervention DCCs		Control DCCs		Difference	Baseline corrected difference*	Baseline corrected†	
	%	(*n*)	%	(*n*)	(%)	(%)	OR	95% CI
**Overall compliance caregivers**	59	(664)	44	(610)	15	25	4·13	2·33–7·32
Eating/food handling	39	(196)	24	(164)	15	14	1·95	0·76–5·00
Before food handling	51	(111)	29	(83)	22	25		
Before caregivers themselves ate	19	(26)	20	(35)	−1	8		
Before caregivers assisted children with eating	25	(59)	20	(46)	5	−19		
Toilet/diapering	73	(322)	57	(272)	16	28	4·49	2·23–9·05
After changing a diaper with faeces	94	(77)	80	(71)	14	20		
After changing a wet diaper when child was lying down	69	(144)	56	(127)	13	26		
After changing a wet diaper when child was standing	56	(79)	31	(65)	25	46		
After wiping buttocks when assisting children with toilet use	82	(22)	78	(9)	4	−6		
Contact with body fluids	55	(105)	38	(127)	17	35	4·88	1·77–13·44
After caregivers coughed/sneezed/wiped their own nose	42	(24)	23	(22)	19	47		
After contact with body fluids	60	(75)	39	(99)	21	39		
Before wound care	33	(3)	33	(3)	n.a.	n.a.		
After wound care	67	(3)	100	(3)	n.a.	n.a.		
After visibly soiled hands	59	(41)	49	(47)	10	11	2·11	0·13–34·22
**Overall compliance children**	38	(605)	41	(475)	−3	−16	0·64	0·18–2·33
Before eating	18	(275)	18	(195)	0	−1		
Before food handling	0	(5)	100	(6)	n.a.	n.a.		
After toilet use	48	(95)	48	(101)	0	−5		
After playing outside	75	(81)	46	(48)	29	3		
After visibly soiled hands	48	(149)	66	(125)	−18	−52		

OR, Odds ratio; CI, confidence interval.; n.a., not applicable (as activities occurred ⩽5 times).

* Difference between intervention and control DCCs at 6 months follow-up minus the difference at baseline.

† Interaction effect of intervention status of the DCC and baseline/follow-up measurement corrected for the type of activities for which HH was indicated, age group (i.e. 0–1, 2–3 and 0–4 years), and clustering of the data within caregivers and within DCCs.

## DISCUSSION

This is the first HH intervention in DCCs developed according to a stepwise behavioural approach targeting the underlying determinants of caregivers' compliance with HH guidelines. To our knowledge, this is also the first study to assess HH compliance of caregivers as primary outcome measure, as well as children's HH compliance. Our study demonstrates that the intervention we developed for DCCs is effective in improving caregivers' compliance with HH guidelines.

Most HH intervention studies in DCCs report as outcome measure the incidence of gastrointestinal and/or respiratory infections, and/or absence of caregivers/children due to illness [11–18]. Although most of these studies show a reduced rate of infection associated with the implementation of the intervention programme, the nature and magnitude of the effect varies (e.g. the effect is not present for both gastrointestinal and respiratory infections) [19]. Insight into a more direct effect of the intervention, namely HH compliance, might explain this, as possibly HH compliance only improved for certain HH indications (e.g. diapering *vs.* nose wiping).

There are few studies to compare our results with. One other DCC intervention study assessed observed HH compliance of caregivers as outcome measure, although no comparison with control DCCs was reported [16]. That study reports that after training, caregivers' HH improved after diapering and after contact with mucus, saliva, vomit, etc. of children [16]. In our study, HH also improved after toilet and diapering activities and after contact with body fluids. The improvement of caregivers' HH after contact with body fluids might be explained by the provision of alcohol-based hand sanitizer which made it possible for caregivers to perform HH after wiping children's noses, e.g. when they were playing outside as some DDCs placed the dispensers by the outdoor storage shed. No effect was found on HH compliance before eating and food-handling activities. Therefore, intervention studies for improving HH compliance in DCCs should pay special attention to these activities.

Another study assessed children's HH behaviour [12]. At 6 months follow-up, the adjusted relative risk for HH before lunch was 2·93 (95% CI 1·86–6·97) and after bathroom use it was 3·30 (95% CI 1·83–16·67) [12]. In two other studies, only compliance of children in intervention DCCs was reported, and no information was given on compliance in control DCCs or at baseline [14, 15, 18]. In our study, we did not find an effect on children's HH compliance. This might be explained by the fact that our intervention primarily focused on caregivers and was developed based on determinants of caregivers' HH behaviour and not children's HH behaviour. Besides the posters and stickers, our intervention did not include components specifically targeting children (e.g. hand-washing songs). Furthermore, our study shows that improving HH compliance of caregivers does not automatically yield improving compliance in supervising children's HH. Determinants of (supervising) children's HH might therefore be different from determinants of caregivers' HH and studies are needed to assess these.

Prior to intervention development, we assessed caregivers' HH compliance in DCCs and showed that the overall compliance was 42% [10]. Although compliance was higher at baseline (i.e. 53% in intervention DCCs and 63% in control DCCs), compliance in control DCCs during follow-up was similar, with little variation over time (43% at T1, 46% at T3, 44% at T6). Because baseline measurement thus seems to be an outlier, especially for control DCCs, we report results both uncorrected and corrected for baseline compliance. At baseline the incidence of gastrointestinal infections was also higher in control DCCs compared to intervention DCCs, which dropped during follow-up [22]. This might explain the high HH compliance in control DCCs at baseline, as our previous qualitative study showed that caregivers usually increase their HH when observing diarrhoea in the children (T. P. Zomer *et al.*, unpublished data).

A strength of our study is that HH compliance of both caregivers and children was observed and that besides overall compliance, the compliance for each of the specific HH indications is also reported. Furthermore, our intervention had multiple components, addressing environmental and sociocognitive determinants of HH. Moreover, exposure to the different intervention components was high, except for the hand cream dispensers that were delivered halfway through the intervention period (because during the team training sessions it became clear that there was a need for hand cream dispensers to reduce sore and dry hands). Other strengths of the study are the randomized controlled design, the high inter-rater reliability among observers, and the large sample size of 71 participating DCCs and 795 observed caregivers. In addition, control DCCs also received the intervention after data collection, which probably facilitated DCC recruitment and minimized loss to follow-up [12].

A possible limitation of our study is the Hawthorne effect; caregivers might change their behaviour when they know they are being observed [27]. Although this bias could not be entirely prevented, it was minimized by observing unobtrusively and by informing caregivers that the focus of the observations was on hygiene in general, not specifically mentioning HH. In addition, the physical appearance of the observers was similar to that of caregivers working in the DCCs, as most of them were young females. Furthermore, repeated exposure to observations could make caregivers less sensitive to adapting their behaviour during observations [26]. Nevertheless, we would expect the Hawthorne effect to be more pronounced in intervention DCCs than in control DCCs, as being exposed to the intervention made it more likely for caregivers to know the purpose of the observation. The intervention effect might then be an overestimation of the true effect size. The incidence of gastrointestinal and respiratory infections in children attending DCCs [22] would then be a more objective outcome measure. Another possible limitation is that observers might have recognized the intervention status of the DCC, which could have biased data collection. Furthermore, children's HH compliance was only assessed in children for which the observed caregiver was responsible, and not in all children in the group. Better assessment of children's HH compliance would include all the children. Another possible limitation is that the participating DCCs also participated in our previous study on determinants of HH behaviour, for which they received information regarding their HH compliance 6 months prior to intervention start. Therefore, the intervention effect might be an underestimation of the true effect size.

In conclusion, this study shows that our intervention, addressing determinants that underlie caregivers' HH behaviour, is effective in improving caregivers' HH compliance in DCCs. Therefore, dissemination of the intervention in other DCCs can be considered (especially when determinants of HH behaviour are similar). DCCs can then implement the intervention to distinguish themselves from a quality perspective from other DCCs. More studies are needed to assess the duration of the intervention effect beyond 6 months and to assess which components of the intervention are most effective.
